# Medical News and Miscellany

**Published:** 1893-04

**Authors:** 


### MARINE HOSPITAL SERVICE.

CIRCULAR.

TREASURY DEPARTMENT,
OFFICE SUPERVISING SURGEON GENERAL.
U. S. MARINE HOSPITAL SERVICE.

WASHINGTON, April 13, 1893.

A board of officers will be convened at Washington, D. C., June 26, 1893, for the purpose of examining applicants for admission to the grade of Assistant Surgeon in the U. S. Marine Hospital Service.

Candidates must be between twenty-one and thirty years of age, graduates of a respectable medical college, and must furnish testimonials from at least two responsible persons as to character.

For further information, or for invitation to appear for examination, address

THE SUPERVISING SURGEON GENERAL,
U. S. Marine Hospital Service, Washington, D. C.

---

**Dr. W. W. Pugh**, of Bryan, writes the JOURNAL that in a number of tedious labors he has found it expedient to place his patient in a large vessel of warm aseptic water, when pains are coming on and the os is dilated. He says the water should come up an inch or so above the umbilicus, and the woman, being in squatting position, labor is facilitated by gravitation, and by getting rid of atmospheric pressure. The water can then be used for bath after delivery of child and percenta, for mother and child. Mother should then be wiped dry and placed in bed. Dr. Pugh claims that by this method sepsis and child-bed fever are prevented; and asks the brethren to give the method a trial.

---

**New Texas Doctors**: The Memphis Hospital Medical College held its Thirteenth Annual Commencement on the 30th of March ult., and graduated a class of ninety, thirty of whom were Texans. This college is a great favorite with Texas boys, and is deservedly popular. Its growth and prosperity have been phenomenal. The new college building, an imposing structure, was "christened" in fine style, and makes the above fine showing. Prof. Sim is is a tower of strength, a whole "Faculty", within himself, not that we would disparage his colleagues by any means.

### Medical News and Miscellany.

Dr. T. D. Wooten, President Board Regents Texas University has been very sick recently. The Journal is glad to announce that he is now able to resume his professional duties.

Dr. Albert Von Chrzasczewski, of Sambos, Galica, Austria, on November 28, 1892, writes. "Bromidia is superior to all other hypnotics, and is free from all unpleasant effects."

Inguinal Hernia.—For next issue we have an excellent article on this subject by Prof. J. E. Thompson M. D. Medical Department University of Texas, which will be illustrated by cuts from pen drawings by the author.

A thoroughly competent druggist, twenty-eight years of age, married, desires a situation. Graduate of pharmacy; speaks Spanish and English and has had ten years experience. Best testimonials as to character habits and qualifications. Address "D", care this Journal, Austin, Texas.

Dr. W. T. Jenkins, the much praised and much abused Port Health Office at New York expresses the opinion—published in an interview recently, that cholera will not get into America. If it could not succeed last year when it took us unawares and caught unprepared, it certainly cannot do so now. It missed the best chance it will ever have.

---

Atlanta Polyclinic.—The Atlanta Polyclinic was formally opened on 22d ult. (March, 1893). The building is admirably adapted for the purposes, and clinical material, both medical and surgical, is abundant. It will be open all the year, and physicians can enter at any time. Southern physicians should show their appreciation of Southern enterprise by a liberal patronage toward this institution. Terms are reasonable and advantages many.

---

The *New York Me*[illegible] *Record* criticises us for "spelling whiskey without an "e"[illegible] We will remark, parenthetically, that Webster spells it like we do, but then, Webster is not *Shrady*, you know; the fact is only mentioned to emphasize that Webster was a *chump*. Now, brother Shrady has had more spells of whisky than we have, and, of course, knows more about it,—we defer to his familiar acquaintance with the subject. He spells it, and doubtless, drinks it, with "e"s.

---

Dr. B. W. Bristow, of Flatonia, has been appointed State Quarantine Officer at Corpus Christi, (Aransas Pass). Dr. J. W. Irion, of Fort Worth, filled the position under Gov. Hogg's former administration. Dr. Bristow is an able physician and a very popular gentleman and will make an excellent quarantine officer. The other stations are filled by the former incumbents, to wit: Dr. Blunt, at Galveston; Dr. Wolff, at Brownsville; Dr. Perkins, at Sabine Pass; Dr. Weisiger, at Velasco, and Dr. Duncan at Pass Cavallo.

---

Died—Dr. J. E. Roach, of Sipe Springs, Texas, died at that place April 10th inst., of acute gastritis. He was a Mason and was buried with Masonic honors. Dr. Roach came to Texas eleven years ago from Atlanta, Georgia, where he was formerly engaged in the practice, and has resided and practiced at Sipe Springs continuously ever since. He was a member of the Texas State Medical Association and stood well with the general pro-ession.

---

Try Salitonia; see advertisement.

### The Therapeutic Merit of Combined Remedies.

BY STEPHEN J. CLARK, M. D., OF NEW YORK, N. Y.

In nearly every case where quinia is indicated, it can be advantageously combined with antikamnia, which thus becomes a valuable adjunct to quinia. Quinia, for example, is a most decided febrifuge, and its action is usually promptly reliable; but when combined with this member of the aromatic series, its action is markedly increased. Some individuals, however, cannot take any of the coal-tar derivatives; consequently the use of antikamnia will be inhibited in such cases; on the other hand, some patients cannot take quinine.

An important benefit to be derived f[illegible] the addition of antikamnia to quinine is that it removes [illegible]nse of fulness in the head, constriction about the forehea[illegible]d tinitus aurium—so common when the latter drug is adm[illegible]tered alone; the disturbing action of quinia on the auditory [illegible]rve is suspended to a great extent, and the usual quinine deafness is absent. The combination of these agents in tablet form is a happy one.

The combination of antikamnia with quinia is valuable in the racking headache, with high fever, attendant upon malarial disorders. It is likewise valuable in cases of periodical attacks of headache of non-defined origin; of the so-called "bilious attack;" of dengue; in neuralgia of the trigomini; in that of "ovarian catarrh;" and, in short, in nearly every case where quinine would ordinarily be prescribed.

Binz claims specific antiseptic powers for quinia; other writers are in accord with him on this point, and report good results from large doses in septicæmia, pyæmia, puerperal fever, and erysipelas. It is a germ destroyer of the bacilli of influenza (la grippe). A full dose of quinine and antikamnia will promptly relieve many cases of this disease. In the gastric catarrh of drunkards this combination is valuable. Quinia is a poison to the minute organism—sarcina; and antikamnia exerts a soothing, quieting effect on the nerve filaments. A full dose of antikamnia and quinia will often arrest a commencing pneumonia or pleuritis. This combination is also useful in the typho-malarial fever of the South—particularly for the hyperpyrexia—both quinia and antikamnia, as previously said, being decided fever reducers.

The germicide power of quinia is the explanation of its success in the treatment of malarial disturbances. Thus it is also a prophylatic against the various manifestations of malarial poison, and as such it can be relied on. The cause of malaria as a disease consists of pigmented bodies, which penetrate the interior of the red blood corpuscles—pigmented bodies of various shapes and flagellate organisms—both having amœboid movements—the filaments being in active vibration.

In meningeal troubles, attended by marked acceleration of the heart due to the rise in the fever temperature, full doses of quinine and antikamnia at intervals of, say, about four hours, will be productive of good. In measles, large doses of the combination at night—say ten grains of each for adults (doses for children in proportion), will relieve the distress of the catrrahal pneumonia, and modify, in a great degree, the amount of the exudative products. The periodical n[illegible]ses which may be either regular or irregular in their manifes[illegible]s, but which are dependent on the malarial germ for their or[illegible]." are all controllable by the combination of quinine and antik[illegible]nia. Examples of such neuroses are asthma, laryngismus s[illegible]idulus, summer catarrh, etc. Indeed, for the hemicrania and neuralgias of malarial origin, the combination of quinine and antikamnia, just alluded to, may be declared *a specific.*

The dose of quinine may be made smaller than usual when administered with antikamnia. Thus, one or two tablets of two and a half grains each of quinine and antikamnia will prove sufficient for great utility in puerperal mania, in the headaches of advanced age, accompanied with vertigo and despondency.

This combination is capable, by the combined influence of each drug on the nervous system and blood, of restraining all the processes which develop heat, organic changes, and muscular motion; therefore, it is "the one thing needful" in the treatment of the hyperpyrexia of malarial fevers. In the vast majority of cases, when necessary to administer quinine, if antikamnia be added to the prescription, the result will be surprising.

Formerly, the idea prevailed that in order to render the treatment of periodical fevers efficient, the gastro-intestinal tube should be cleaned out by emetics and cathartics. This, however, is a fallacy, as the conditions they are intended to remove depend mainly on the malarial poison, for which the combination of quinine and antikamnia is the specific cure.

In speaking of the treatment of pneumonia by quinine and antikamnia, Prof. Palmer says: "The effects desired, and certainly as a rule produced, are a decided reduction of temperature, a marked diminution in the frequency of the pulse, a decided moisture of the skin or free sweating, a slower and more easy respiration, or relief from pain, and the feeling of fullness of the chest, a diminution of the cough and of the tenacious and bloody character of the expectoration; and, in short, not only is there a checking of the fever, but of all evidences—general and local—of the pulmonary engorgement and inflammation."

In Meniere's disease, or "labyrinthine vertigo," this combination has, by persistent use, entirely removed the trouble in many cases. The curative effects of quinine and the coal-tar antipyretics in sun stroke are well known, and have been used recently with great benefit in numerous instances in this country and in India. In hysteria, and even in epilepsy, the combination of quinine and antikamnia is often indicated, and will frequently give the desired results. In whooping-cough and hay fever, quinine and antikamnia will prove beneficial.

The tablets of equal parts of quinine and antikamnia, spoken of in this article, can be administered by the rectum, with good effect. They should first be dissolved in whiskey, and then water can be added in any quantity needed—always remembering the total quantity of each drug in such enemata.

*66 West Tenth Street.*

